# Ground Tire Rubber Filled Flexible Polyurethane Foam—Effect of Waste Rubber Treatment on Composite Performance

**DOI:** 10.3390/ma14143807

**Published:** 2021-07-07

**Authors:** Paulina Kosmela, Adam Olszewski, Łukasz Zedler, Paulina Burger, Adam Piasecki, Krzysztof Formela, Aleksander Hejna

**Affiliations:** 1 Department of Polymer Technology, Gdańsk University of Technology, Narutowicza 11/12, 80-233 Gdańsk, Poland; paulina.kosmela@pg.edu.pl (P.K.); adam.olszewski@student.pg.edu.pl (A.O.); lukasz.zedler@pg.edu.pl (Ł.Z.); paulina_anna_burger@interia.pl (P.B.); krzform1@pg.edu.pl (K.F.); 2 Institute of Materials Engineering, Poznan University of Technology, Piotrowo 3, 61-138 Poznań, Poland; adam.piasecki@put.poznan.pl

**Keywords:** polyurethane foams, ground tire rubber, composites, oil modification, recycling

## Abstract

The application range of flexible polyurethane (PU) foams is comprehensive because of their versatility and flexibility in adjusting structure and performance. In addition to the investigations associated with further broadening of their potential properties, researchers are looking for new raw materials, beneficially originated from renewable resources or recycling. A great example of such a material is ground tire rubber (GTR)—the product of the material recycling of post-consumer car tires. To fully exploit the benefits of this material, it should be modified to enhance the interfacial interactions between PU and GTR. In the presented work, GTR particles were thermo-mechanically modified with the addition of fresh and waste rapeseed oil in the reactive extrusion process. The introduction of modified GTR particles into a flexible PU matrix caused a beneficial 17–28% decrease in average cell diameters. Such an effect caused an even 5% drop in thermal conductivity coefficient values, enhancing thermal insulation performance. The application of waste oil resulted in the superior mechanical performance of composites compared to the fresh one and thermo-mechanical modification without oils. The compressive and tensile performance of composites filled with waste oil-modified GTR was almost the same as for the unfilled foam. Moreover, the introduction of ground tire rubber particles enhanced the thermal stability of neat polyurethane foam.

## 1. Introduction

Polyurethanes (PU) are very versatile materials with a broad range of potential industrial applications. Therefore, the PU market is constantly growing over the last decades [1]. Currently, the global demand for polyurethane materials is estimated at around 20.4 million tons [2]. Forecasts indicate that in 2024 it should increase by around 10% and reach 22.5 million tons [3]. Among all polyurethane materials, 59% accounts for the polyurethane foams, while 31% solely for the flexible polyurethane foams [4,5]. They are commonly applied in the furniture, automotive, construction, packaging industries, as well as damping and soundproofing materials [6]. The growth of the polyurethane market poses many challenges for manufacturers but, at the same time, an exciting opportunity. Their activities are focused on improving current products, expanding their offer, and increasing production profitability. Among the main directions of development of flexible polyurethane foams, also pronounced by the producers, should be mentioned increasing the functionality, reducing waste generation, or reducing materials’ costs, e.g., by applying the recycled raw materials [7]. The use of recycled raw materials in polyurethane technology can be realized using recycled polyols or the introduction of fillers. Considering polyols, they can be obtained, e.g., by glycolysis of polyurethanes, or poly(ethylene terephthalate), or by liquefying lignocellulosic biomass [8,9,10]. Solutions based on the use of recycled polyols are already present in the polyurethanes market. Manufacturers offer products derived from glycolysis processes waste polyurethane foams or glycolysis of waste poly(ethylene terephthalate) from used bottles [11,12,13]. Nevertheless, it is still entirely reasonable to search for further possibilities of using recycled materials to produce polyurethanes, including foams.

Therefore, except for recycled polyols, various waste-based fillers may be introduced into a foamed polyurethane matrix. One of the possibilities is to use wastes generated during polyurethane foam production and prepare all-polyurethane composites [14]. Such solutions are currently present in the market and used as carpet linings or floor underlays [15]. Among the other potential fillers for the flexible polyurethane foams could be mentioned waste rubber particles such as ground tire rubber (GTR). It is the material generated during material recycling of post-consumer car tires [16]. As mentioned above, the multiple applications of flexible PU foams include the products, whose important aspect is the material cost. Therefore, the use of relatively cheap GTR could reduce the cost of the material and significantly increase its attractiveness to potential buyers [17]. Ground tire rubber is significantly cheaper than commonly applied polyurethane systems [18]. Except for the cost benefits, the introduction of waste rubber may enhance the performance of flexible polyurethane foams. Literature data indicate that it may beneficially affect the compressive strength and the damping properties considering the mechanical and acoustic vibrations [19]. Such effects play a vital role in the applications of PU foams in packaging, automotive, and construction industries or as soundproofing materials [20]. Gayathri et al. [21] observed a substantial enhancement of tensile and compressive performance of foams with the addition of waste rubber. The strength of the material was increased by over 100% for the 2 wt% filler addition. Similar effects associated with the strength increase were noted by Cachaço et al. [22]. Moreover, Gayathri et al. [21] reported the significant rise of the sound absorption coefficient of foams after GTR addition, which is a great advantage from the application point of view. Depending on the applied sound frequency, the absorption was even 60% higher than for unfilled foam. A similar enhancement of the soundproofing performance was reported by Zhang et al. [23]. They investigated the impact of unmodified and partially devulcanized GTR. Modification was performed using pan-mill type mechanochemical reactor at ambient temperature. It enabled reduction of particle size from ~250 µm to ~60 µm. Moreover, the X-ray photoelectron spectral analysis indicated the 3.4% increase of the oxygen content, indicating partial oxidation of the GTR surface. Such an effect was beneficial for the foams’ cellular structure and enabled reduction of the average cell diameter, pointing to the enhanced interfacial interactions with polyurethane matrix. Such an effect is very beneficial for the foams’ performance [24]. Composites filled with 20 wt% of neat and devulcanized GTR showed the sound absorption coefficients (at 1000 Hz) of 0.143 and 0.242. Moreover, the loss modulus determined by the dynamic mechanical analysis was higher for application of modified GTR over the whole range of analyzed frequencies (from 0 to 180 Hz). The beneficial effect of devulcanization was also observed for higher filler loadings. For the 30 wt% content, the absorption coefficient around 1000 Hz reached even the value of 0.350, which was attributed to the enhanced elasticity of foams. Presented results show that the partial devulcanization of GTR is very promising approach for the manufacturing of PU/GTR based damping materials. Nevertheless, our previous results [19] indicate that after modifications of GTR, the formulations of PU foams should be modified to adjust the proper ratio between isocyanate and hydroxyl groups in the system. Such a phenomenon was confirmed by our other work [20] dealing with the application of GTR oxidized with KMnO_4_ and H_2_O_2_ solutions. An excessive oxidation of GTR surface with potassium permanganate caused the disturbance of the NCO:OH ratio, which resulted in the weakening of polyurethane matrix and significant reduction in compressive strength. On the other hand, the results for composites filled with H_2_O_2_ modified GTR were very promising. Summing up, the literature works indicate that the proper modification of ground tire rubber should be considered auspicious for the performance of foamed PU/GTR composites. However, more different approaches should be investigated.

Keeping in mind the potentials benefits of the GTR introduction into a foamed flexible polyurethane matrix, we also investigated the application of this waste as a filler in the presented work. As suggested by the works mentioned above, to enhance the interfacial interactions between the PU matrix and GTR, prior to the introduction, the filler was modified. Thermo-mechanical treatment in the co-rotating twin-screw extruder was applied. Such a process is very efficient in terms of GTR surface modification [25]. The impact of GTR treatment on the cellular structure, physical, thermal, as well as static and dynamic mechanical performance was investigated. Moreover, for more efficient surface activation and partial swelling of GTR particles, the two types of rapeseed oil were introduced as additional modifiers—fresh and waste oil, obtained from the local restaurant. Typically, the waste rapeseed oil differs from the fresh one due to the number of chemical changes occurring during cooking, e.g., hydrolysis, oxidation, oligomerization, as well as the extraction of chemical compounds from food products [26]. As a result, waste oils are characterized by the higher acid values and lower iodine values [26]. Moreover, oxidation of oils occurring during primary use can be confirmed by the significantly higher peroxide values.

## 2. Materials and Methods

### 2.1. Materials

The materials used in the presented study are listed in Table 1.

### 2.2. Modifications of Ground Tire Rubber

**Figure 1:**
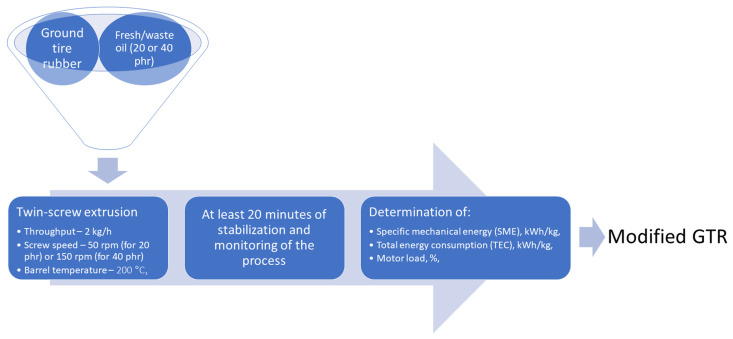
The scheme of GTR modifications performed in the twin-screw extruder.

Treatment of GTR was performed with an EHP 2 × 20 Sline co-rotating twin-screw extruder from Zamak Mercator (Skawina, Poland) as described in our previous work [27]. More details are presented in Figure 1. For GTR modified with 20 phr of oils, the screw speed of 50 rpm was the minimum speed enabling efficient modification. For samples containing 40 phr of oils, the screw speed had to be increased to 150 rpm because of clogging in the dosing section. For comparison, thermo-mechanically modified GTR without oil addition was also analyzed.

Table 2 presents the properties of modified GTR samples, including the hydroxyl values (L_OH_) determined according to the method based on the modified test method for isocyanate groups, as described in our previous works [28,29]. Hydroxyl values were calculated based on the free isocyanate group content (%_NCO_) in GTR:isocyanate mixtures and the differences in %_NCO_ between the mixture and neat isocyanate (Δ_NCO_). For comparison, the hydroxyl value of neat ground tire rubber applied in the presented study equaled 61.7 mg KOH/g. Moreover, the specific mechanical energy (SME) values and total energy consumption (TEC) are presented.

As presented in our previous works [27,28,29], thermo-mechanical treatment of the ground tire rubber in twin-screw extruder may result in chemical changes on the surface of particles, especially partial oxidation and generation of hydroxyl, formyl and carbonyl groups. The oxidative degradation of GTR resulting in the generation of formyl and carbonyl groups was reported by Gągol et al. [30]. Such an effect is attributed to the high shear forces acting on the material during twin-screw extrusion and the atmosphere of the process—air, which enables oxidation of material. Formela et al. [16] proved that even the short treatment of GTR in the extruder can result in an appearance of hydroxyl groups on the surface of analyzed waste material (even when the process is conducted at only 120 °C). As mentioned above, the GTR samples used in the presented work were modified at a significantly elevated temperature of 200 °C under the air atmosphere. Multiple research works reported that thermal decomposition of GTR in the air begins around 200 °C [31,32,33].

Moreover, the whole process (extrusion with specific screws configuration) generates shear forces adding additional energy to the system. In the work of Zedler et al. [34], research was conducted on extrusion of modified rubber. The temperature settings of the extruder were as follows: 40/40/60/60/60/60/60/60/60/60/60 °C. Temperature measurements were conducted on the extruder nozzle using a thermal imaging camera. The obtained thermograms indicated that the temperature increased more than two times compared to the extruder heating zone settings. This phenomenon only indicates the significant influence of shear forces generated by appropriately selected screws segments, on changes in the energy of the system. Therefore, thermal analysis methods conducted on a small sample, under static mechanical conditions and the presence of inert gas, cannot be fully compared to the actual stability of material processed in an extruder generating high shear forces and with access to air.

Generally, thermo-mechanical treatment under proposed conditions results in oxidation of rubber and generation of functional groups, including hydroxyls. Such an effect was also noted by Zhang et al. [35], who noted significant increase in the oxygen content in GTR after milling at ambient temperature. Similar effects were reported in other works [36,37]. As a result, the analyzed samples of GTR shown hydroxyl values exceeding 30 mg KOH/g.

### 2.3. Preparation of Polyurethane/Ground Tire Rubber Composite Foams

Samples were prepared on a laboratory scale by a single-step method. Introduced filler was mixed with the applied polyols at 1000 rpm for 60 s to guarantee its proper distribution. Then, all components were mixed for 10 s at 1800 rpm and poured into a closed aluminum mold with dimensions of 20 × 10 × 4 cm^3^. All analyses were performed after 24-hour conditioning of samples at room temperature and average humidity of 60%. In the following sections, the neat foam without the addition of GTR was named PU. In contrast, composite foams were named GTR_X_, where X indicates the type and content of introduced GTR. Table 3 shows the formulations of prepared composite foams. All foams were characterized by a similar level of apparent density—205 ± 6 kg/m^3^.

### 2.4. Characterization Techniques

After conditioning, foamed polyurethane composites were cut into samples whose properties were later determined following the standard procedures.

The samples’ morphology was evaluated using a scanning electron microscope (SEM) MIRA3—produced by the Tescan (Brno, Czech Republic). The thin carbon coating with a thickness of approximately 20 nm was deposited on samples using Jeol JEE 4B vacuum evaporator from Jeol USA (Peabody, MA, USA). The cellular structures of foams were analyzed using an accelerating voltage of 5 kV. The secondary electron detector was used.

The images obtained with the SEM microscopy were analyzed with ImageJ software. Except for the average cell diameter, the following shape descriptors of cells were determined:

Aspect ratio (AR) calculated according to the following Equation (1):

AR = L_L_ / L_S_ (1)

Roundness (R) calculated according to the following Equation (2):

R = (4 · A) / (π · L_L_^2^) (2)

where: L_L_ and L_S_—the lengths of the longer and shorter axis of the fitted ellipse; A—the area of fitted ellipse.

At least 150 cells for each sample were taken into account during analysis.

The content of open cells in foamed composites was determined using an Ultrapyc 5000 Foam gas pycnometer from Anton Paar (Graz, Austria). Following measurement settings were applied: gas—helium; target pressure—3.0 psi; foam mode—on; measurement type —corrected; flow direction—sample first; temperature control—on; target temperature—20.0 °C; flow mode—monolith; cell size—medium, 45 cm^3^; preparation mode—flow; time of the gas flow—0.5 min.

The thermal conductivity coefficient (λ) or prepared polyurethane foams was determined using the heat flow meter HFM 446 from Netzsch (Selb, Germany). Samples with thickness of 4 cm were tested in the temperature range from 1 to 19 °C using the average temperature of 10 °C.

Sol fraction content was determined as the mass difference of prepared foams before swelling in xylene (W_1_) and after extraction (W_2_), according to the following Equation (3):

Sol fraction content = (W_1_ − W_2_) / W_1_ · 100% (3)

The compressive strength of studied samples was estimated following ISO 604. The cylindric samples with dimensions of 20 mm × 20 mm (height and diameter) were measured with a slide caliper with an accuracy of 0.1 mm. The compression test was performed on a Zwick/Roell Z020 tensile tester (Ulm, Germany) at a constant speed of 15%/min until reaching 60% deformation.

The tensile strength of microporous polyurethane elastomers was estimated following ISO 1798. The beam-shaped samples with 10 × 10 × 100 mm^3^ dimensions were measured with a slide caliper with an accuracy of 0.1 mm. The tensile test was performed on a Zwick/Roell tensile tester at a constant speed of 500 mm/min.

Dynamical mechanical analysis (DMA) was performed using a Q800 DMA instrument from TA Instruments (New Castle, DE, USA) at a heating rate of 4 °C/min and the temperature range from −100 to 150 °C. Samples were cylindrical-shaped, with dimensions of 10 × 12 mm.

The thermogravimetric (TGA) analysis of GTR and composites was performed using the TG 209 F3 apparatus from Netzsch. Samples of foams weighing approx. 10 mg were placed in a ceramic dish. The study was conducted in an inert gas atmosphere—nitrogen in the range from 30 to 800 °C with a temperature increase rate of 10 °C/min.

## 3. Results and Discussion

**Figure 2:**
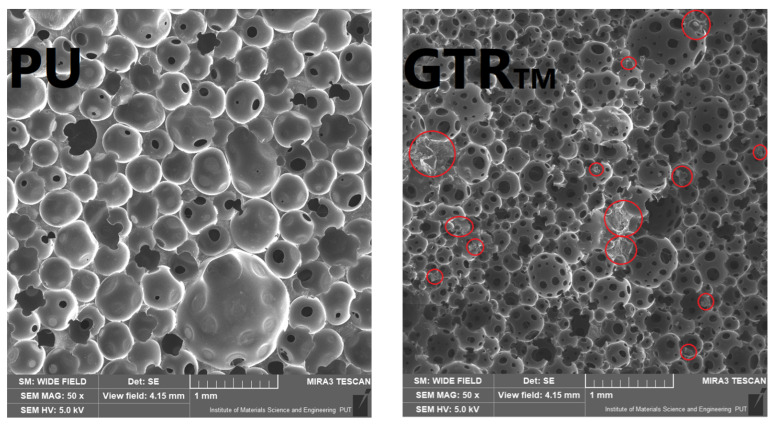

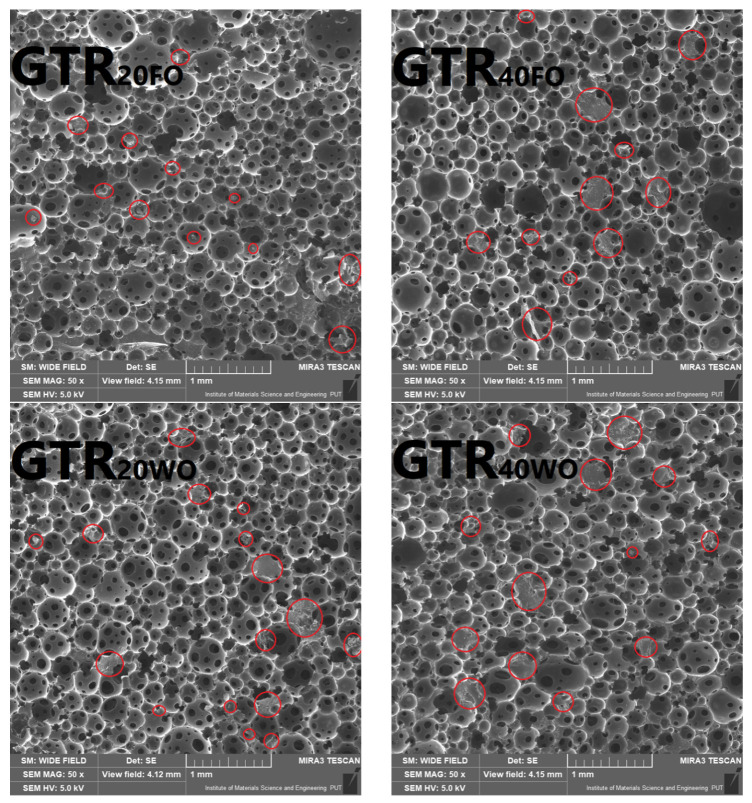
The SEM images showing the cellular structure of prepared foams (GTR particles marked with red circles).

The cellular structure of prepared foams is presented in Figure 2. Moreover, the parameters describing the structure are summarized in Table 4. The changes in the cellular structure of polyurethane foams are related to the changes in the reaction mixture’s viscosity caused by introducing solid rubber particles, as reported by Paberza et al. [38]. In their work, the viscosity of the polyol mixture was exponentially rising with the addition of solid lignin particles from the initial 3.0 Pa·s to 24.2 Pa·s for the 17.5 wt% filler loading. Moreover, solid particles may act as nucleating agents in polyurethane systems [39]. Previous works [40,41] pointed to reducing the nucleation free energy, which favors the formation of nucleation sites and increasing the number of cells in the foams’ structure. Lee et al. [40] indicated that the nucleating effect of solid particles depends on their size, shape, and compatibility with the polyurethane system, which affects the surface tension. High compatibility results in the more significant reduction of free energy and increases the number of nucleation sites, reducing the average particle size.

It can be seen that the introduction of the ground tire rubber particles resulted in a noticeable decrease in average cell diameter from 308 µm to 220–257 µm, depending on the rubber treatment. Such an effect was also noted in our previous work [42]. This effect was associated with the increase in the polyol mixture’s viscosity, which, as mentioned above, affects the foaming kinetics [43]. For oil-modified GTR particles, the cell size reduction was higher than for thermo-mechanically modified rubber due to the increase in surface tension caused by the oil presence [44]. As a result, more energy was required to form and especially grow the cells during the polymerization of the polyurethane matrix. Moreover, the presence of oil during extrusion treatment of GTR at elevated temperature may result in swelling of rubber particles leading to enhanced interfacial interactions with polyurethane matrix [45].

Except for the physical effects of GTR oil modification on the foams’ cellular structure, the chemical interactions at the interface have to be considered. As presented in Table 2, the applied samples of GTR were characterized by the varying hydroxyl values, pointing to the different content of hydroxyl groups present on the rubber surface. As presented in our previous works [27,28,29], the oil-assisted thermo-mechanical treatment caused changes in the GTR surface structure. When the fresh oil was applied, the hydroxyl value was reduced, attributed to the chemical structure of rapeseed oil, especially oleic, linoleic, α-linolenic, palmitic, and stearic acids which are the main fatty acids present in this oil [46]. These acids do not contain hydroxyl groups in the structure, so they are not contributing to the hydroxyl number [47]. As a result, the fresh rapeseed oil was only swelling the GTR particles without introducing additional functional groups.

Nevertheless, after frying, the chemical structure of oils changes due to their hydrolysis, oxidation, and polymerization [26]. The first two groups of reactions may contribute to the hydroxyl number of modified GTR. The hydrolysis results in the formation of mono- and diglycerols, free fatty acids, and sometimes even glycerol, which was also noted in other works [26]. These compounds contain hydroxyl groups so that they can increase the hydroxyl value of modified GTR. Oxidation of oils may result in the generation of carbonyl groups, noticeably less often hydroxyls. Nevertheless, it may also slightly affect the hydroxyl number of oil [48]. Therefore, the hydroxyl values of waste oil-modified GTR were noticeably higher compared to the other samples. As a result, the interactions with polyurethane matrix were enhanced, which resulted in a slightly more substantial reduction of particle size than the samples modified with fresh oil or without oil [40].

Except for the particle size reduction, the introduction of neat and modified GTR particles caused a slight increase in cells’ aspect ratio and reduced their roundness. Such an effect was attributed to the increased viscosity of polyol mixtures containing solid rubber particles leading to the higher heterogeneity of the structure, as indicated by Song et al. [49]. A similar increase of cells’ anisotropy was observed in our other work [50]. The reduction of the average cell diameter as a function of increasing polyol viscosity was also reported by Fan et al. [51]. They attributed this effect to the limited coalescence among gas bubbles, which were not merging during the volumetric expansion of the material [52].

Considering the open cell content, the critical parameter of cellular materials, the influence of GTR introduction was minimal, confirming the results presented in our previous paper [42]. A slight decrease in the content of open cells can be attributed to the above-mentioned increase of polyols’ viscosity and closing of cells due to the reduced coalescence [53]. On the other hand, the presence of filler particles may sometimes increase the content of open cells [54]. As a result of the combined impact of GTR particles, open cell content in the presented samples was hardly affected. A similar phenomenon related to the filler incorporation into flexible polyurethane foams was noted by Javni et al. [55].

The changes in the cellular structure caused by the introduction of GTR particles into a flexible foamed polyurethane matrix impacted its thermal conductivity. Generally, this property is rather associated with rigid polyurethane foams, which are one of the most popular thermal insulation materials [56]. Nevertheless, flexible foams are also used, e.g., in building applications as floor underlays, where thermal conductivity coefficient (λ) is quite an important parameter [57]. In the case of cellular materials, thermal insulation properties are directly associated with their morphology. According to Szycher [58], the thermal conductivity coefficient of foamed materials can be described by the following Equation (4):

λ = λ_solid_ + λ_gas_ + λ_convection_ + λ_radiation_ (4)

For analyzed materials, the value of λ coefficient attributed to solids is affected by the introduction of GTR into the polyurethane matrix. Depending on the literature reports, the λ value of shredded tire rubber varies between 0.100 and 0.166 W/(m·K) [59,60]. For the nonporous polyurethane, thermal conductivity coefficient is in the range of 0.200–0.260 W/(m·K), depending on the applied formulation [61,62]. Therefore, simple replacement of some portion of solid polyurethane in foam should guarantee the reduction of its thermal conductivity coefficient. Nevertheless, the introduction of GTR often results in the disruption of cellular structure, which was shown in our previous papers [19,63]. Moreover, it can be seen that the application of the oil-modified GTR resulted in higher values of thermal conductivity coefficient compared to the sample GTR_TM_. Such an effect could be attributed to the higher thermal conductivity of rapeseed oil comparing to the rubber, which is around 0.170–0.180 W/(m·K) [64].

Considering the λ_gas_, it is directly associated with the apparent density of cellular materials, which describes the share of solid material in a given volume of foam. Therefore, foams having relatively low apparent density are usually preferred for thermal insulation materials. The typical apparent densities of rigid polyurethane foams or expanded polystyrene applied as insulations range from 30–45 kg/m^3^ [65]. Moreover, the value of λ_gas_ can be influenced by the selection of a proper foaming agent. According to Randall and Lee [66], the application of conventional hydrofluorocarbon as a physical blowing agent instead of the chemical foaming with the carbon dioxide generated in the water reaction with isocyanates may reduce the λ_gas_ by around 50%. At the same time, the thermal conductivity coefficient of CO_2_ is almost 40% lower than air (15.3 vs. 24.9 mW/(m·K)) [66]. Therefore, λ_gas_, and in particular, its stability, is strongly affected by the closed cell content in foam. When the CO_2_ or physical blowing agents are trapped inside closed cells, their diffusive exchange with air is significantly slower. Nevertheless, for presented foams, the impact of λ_gas_ on the total value of thermal conductivity coefficient is similar for all samples due to the similar level of apparent density—205 ± 6 kg/m^3^.

The content of open and closed cells also influences the λ_convection_. The convection can be generally described as the spontaneously occurring fluid flow caused by the combined effects of its heterogeneity and the external factors [67]. Considering thermal conductivity, it is attributed to the gas displacement caused by the temperature gradient in a given volume [53]. It can be quantified with the following Equation (5):

q = h · A · ΔT(5)

where: q—the amount of heat transferred per unit time, W; h—the convective heat transfer coefficient, W/(m^2^·K); A—the heat transfer area, m^2^; ΔT—temperature gradient implicating convection, K.

Therefore, the convection is proportionally affected by the heat transfer area, which is depending on the content of closed and open cells inside the foam. The increasing content of open cells significantly increases the convection area. When the high closed cell content characterizes foams, the convective heat transfer can even be omitted [68]. As shown in Table 4, the introduction of GTR particles into foamed polyurethane matrix caused the decrease in open cell content from 84.7% to 83.1–84.3%. Therefore, the heat transfer area was slightly limited, but the effect was minimal and could be neglected.

The last component of the thermal conductivity coefficient is associated with the radiation heat transfer. According to Glicksman [62], it can account for around 20–30% of the total heat transfer for low-density foams. Over the years, researchers tried to provide the mathematical formula quantifying the radiative heat transfer with different concepts related to the contribution of particular foam components, e.g., cell walls and struts [69,70,71]. Generally, the λ_radiation_ can be determined using the following Equations (6) and (7) [62]:

λ_radiation_ = (16 · σ · T^3^) / (3 · K) (6)

where:

K = 4.1 · ((f_s_ · ρ_f_ / ρ_p_)^0.5^) / d (7)

where: σ—Stefan-Boltzmann constant, 5.67·10^−8^ W/(m^2^·K^4^); T—temperature, K; K—Rosseland mean extinction coefficient depending on foam geometry and material properties, cm^−1^; f_s_—polymer fraction in struts; ρ_f_—density of foam, kg/m^3^; ρ_p_—density of polymer, kg/m^3^; d—cell diameter, m.

Considering the presented equations, the λ_radiation_ is proportional to the cell diameter, so the thermal insulation performance of foam can be enhanced by reducing cell size. The multiple experimental works confirmed such an assumption. Kurańska et al. [72] showed that the thermal conductivity coefficient of polyurethane foam was increased by 5% when the average cross-section area of cells was increased by 5% with the closed cell content maintained at a similar level. In other work, Randall and Lee [66] indicated that the increase in average cell diameter from 0.25 to 0.60 mm implicated the 50% rise of foam’s λ coefficient. In our previous works [50,53], we showed that the 24–25% drop of cell size resulted in the λ reduction by 7–12%, depending on the applied foam formulation. 

The introduction of ground tire rubber particles into flexible polyurethane matrix implicated the decrease in average cell diameter from 308 µm to 220–257 µm. Such an effect is very beneficial for the thermal insulation performance of foams, as proven by the equations mentioned above. As a result, the thermal conductivity coefficient of the reference polyurethane foam was reduced by the incorporation of GTR, irrespectively of the applied treatment. Observed λ changes have to be considered very beneficial because the incorporation of 20 wt% of waste filler enables enhancement of foams’ thermal insulation performance. It is worth mentioning that such an effect is not typical because the introduction of filler causes deterioration of the thermal insulation properties of polyurethane foams [73,74,75].

Table 5 presents the physico-mechanical properties of prepared polyurethane foams. It can be seen that the introduction of GTR, irrespectively of its type, caused a significant increase in the sol fraction content, which for the unfilled foam, equaled 2.3%. Such a low value points to the efficient polymerization of the system and a low portion of unbound extractives [76]. Higher sol fraction contents for composite foams are attributed to the noticeably higher value of this parameter for GTR itself (even over 10% [34,77]), as well as to the GTR interactions with the polyurethane matrix. The functional groups present on the surface of rubber particles (see GTR hydroxyl values in Table 2) may interact with isocyanates present in the polyurethane system, resulting in partially loose polyols macromolecules [78].

Incorporating GTR modified with the fresh rapeseed oil resulted in a more substantial increase of the sol fraction content. It can be attributed to the chemical composition of rapeseed oil and the lack of hydroxyl groups in the structure of fatty acids [46]. As mentioned above, it results in the lower hydroxyl value of modified GTR compared to thermo-mechanical treatment. The fresh oil is hardly bound to the rubber surface or polyurethane matrix so that it can be easily removed during swelling with xylene. On the contrary, the use of waste oil caused a slight decrease in the sol fraction content. It may indicate the enhanced crosslinking of foams’ structure compared to the GTR_TM_ sample. Such an effect was probably related to the above-mentioned changes in the oil structure during frying, such as hydrolysis, oxidation, and polymerization. Compared to the fresh oil, the interactions with the polyurethane matrix were enhanced, so a smaller portion of GTR and oil was removed during swelling.

**Figure 3:**
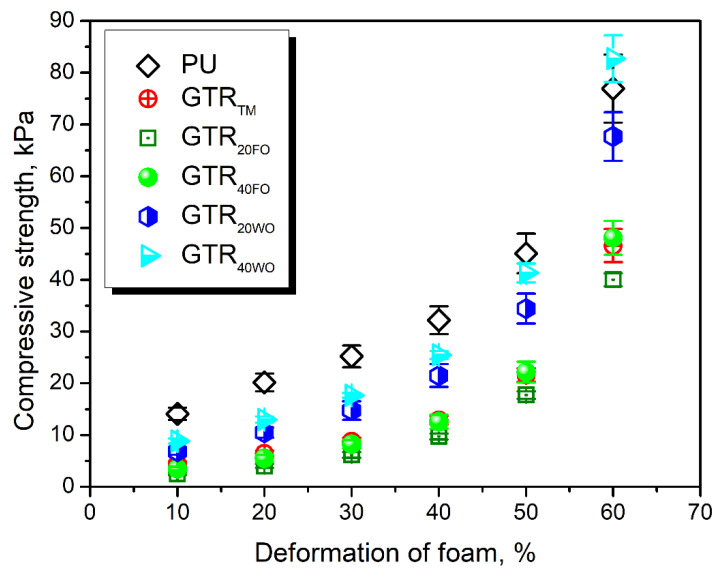
Compressive strength of prepared foams at varying deformation.

Figure 3 presents the compressive performance of analyzed foams. Clearly, the introduction of GTR into neat polyurethane foam caused a reduction of its compressive strength. A similar effect was noted in our previous works [19,42]. It points to the insufficient interfacial interactions between thermo-mechanically treated GTR and PU matrix. The application of fresh rapeseed oil as a GTR modifier resulted in a further decrease in foams’ compressive strength, which could be associated with reducing their stiffness, as suggested by the values of sol fraction content. Deterioration was noted despite the small but beneficial changes in the foams’ cellular structure (see Table 4). In polyurethane foams, the decrease in average cell size and closing of cells is beneficial for the compressive strength since it results from the buckling of cell walls and structure densification [79,80]. The use of waste oil improved the compressive performance of foams compared to the GTR_TM_ sample, which points to the effective swelling of rubber particles and activation of their surface, enhancing the interfacial interactions.

Interestingly, at 60% deformation, the difference in the compressive performance of unfilled foam and composites containing GTR particles was lower. Such an effect could be attributed to the densification of structure. For neat foam, without the solid particles, the densification was not complete, and foam could still reduce its thickness. For composite foams, this phenomenon occurred at lower deformations because of the difference between the average particle size of applied GTR (~0.6 mm) and the average cell size of foams (between 0.22 and 0.31 mm).

Considering the tensile properties, incorporation of GTR, irrespectively of its type, caused performance deterioration. Like the compressive performance, the deterioration suggests the insufficient strength of the interfacial interactions in prepared composites. It was particularly pronounced when GTR was treated with the fresh rapeseed oil, despite the drop in average cell size, which promotes the tensile strength by the facilitated stress distribution [81]. Significant deterioration of the mechanical performance was noted compared to the GTR_TM_ material. Such an effect can be attributed to the noticeably higher values of sol fraction content, which may suggest the reduced crosslink density of the material. As mentioned above, the fresh rapeseed oil contains hardly any hydroxyl groups, which could contribute to the crosslinking of foams, expressed by the relatively low hydroxyl values of GTR modified with fresh oil (see Table 2) [46].

On the contrary, the modification of GTR with the waste rapeseed oil improved the tensile performance of composite foams, attributed to the structural changes in foams. Compared to the GTR_TM_ foam, composites containing waste oil were characterized by ~8 and ~14% smaller cells and lower values of sol fraction content. According to the literature data, such changes are very beneficial for the mechanical performance of cellular materials [79].

**Figure 4:**
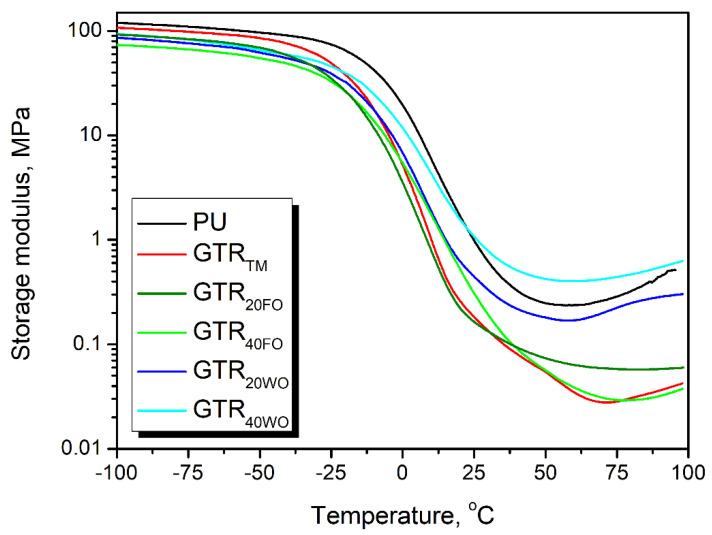
The temperature plot of storage modulus of prepared foams.

For a more detailed analysis of the composite foams’ mechanical performance, the dynamic mechanical analysis was performed. It revealed that the changes in the compressive and tensile performance of prepared foams were directly associated with their glass transition. In Table 5, there are presented values of foams’ glass transition temperatures (T_g_). They were determined as the positions of peaks on the temperature plot of loss tangent (tan δ), also called the damping factor. Polymer materials undergo significant structural changes around the T_g_, which affect their mechanical performance [82]. It can be seen that the ambient temperature, at which the mechanical tests were conducted (22 °C), is close to the T_g_ of prepared foams (13.0–20.7 °C). Figure 4 shows, on the example of storage modulus, that the changes in the mechanical properties of foams take place in the noticeably broader temperature range than just T_g_ [83]. As a result, the closeness of T_g_ significantly affects the static and dynamic mechanical performance of foams. According to the work of Hatakeyama et al. [84], the differences in the glass transition temperature of flexible polyurethane foams in the range of 0–20 °C may cause the significant, even 40%, changes of compressive strength. Presented results confirmed this phenomenon because foams with lower T_g_, hence the bigger difference between T_g_ and temperature of tensile and compression tests, were characterized by the lower strength.

**Figure 5:**
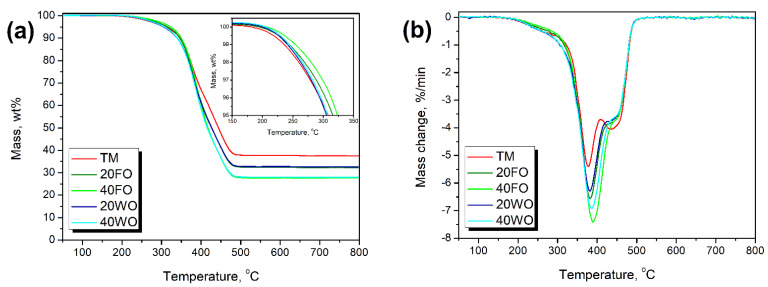
Temperature plots of (**a**) mass loss and (**b**) mass loss rate of applied GTR samples.

Figure 5 and Table 6 present the results of thermogravimetric analysis of applied GTR fillers. The onset of the TM sample, determined as the temperature corresponding to the 2 wt% mass loss, was 260.0 °C. The course of thermal decomposition of GTR was attributed to its composition and content of natural rubber and styrene-butadiene rubber. The first one decomposes at lower temperatures, with the maximum rate around 375–390 °C (T_max1_), while the second one at higher temperatures—430–445 °C (T_max2_) [85].

The oil modification enhanced the stability of rubber particles compared to thermo-mechanical treatment. The introduction of the fresh rapeseed oil shifted the onset towards higher temperatures by 9.3 and 20.2 °C, for 20 and 40 phr loading, respectively. The enhancement was also noted for the waste oil application, but the shift was noticeably lower. Such differences are related to the high stability of rapeseed oil and its partial decomposition during the primary use in gastronomy [42]. Laza and Bereczky [86] reported that thermal degradation of fresh rapeseed oil occurs within the temperature range of 325–500 °C with a maximum rate of around 440 °C. On the other hand, cooking of oil causes its partial decomposition and results in lower-molecular weight compounds, which are often characterized by lower thermal stability [26].

**Figure 6:**
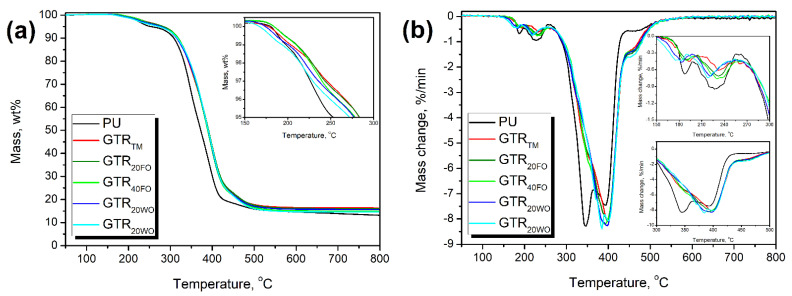
Temperature plots of (**a**) mass loss and (**b**) mass loss rate of prepared foams.

Figure 6 demonstrates the results of thermogravimetric analysis of prepared composite foams. The decomposition of polyurethane foam occurs in the temperature range of 200–500 °C with only minor mass loss at higher temperatures, typical for the flexible polyurethane foams [20]. Thermal stability of the reference foam, determined as the temperature of 2 wt% mass loss, was enhanced after the introduction of GTR particles. Only for the GTR_40WO_ sample, the stability was maintained at a similar level. Noticeably more significant improvement was noted for 5 wt% mass loss. It was attributed to the higher stability of rubber particles compared to the unfilled polyurethane foam. 

Generally, the decomposition of the unfilled PU foam showed five main steps, which are associated with the segment structure of polyurethane [87]. The steps with the maximum rate at 182–194°C (T_max1_) and 218–233 °C (T_max2_) can be attributed to the dissociation of urethane bonds [88]. The presence of two peaks is related to the applied formulation of foams and the use of two different polyether polyols and the glycerol, which led to the generation of structurally different hard segments [89]. The low magnitude of these peaks is associated with the low value of the isocyanate:hydroxyl ratio applied during preparation of foams and the lack of isocyanate’s excess, which could enhance the content of the hard segment. The following signals in the temperature range of 300–420 °C (T_max3_ and T_max4_) are characteristic for the decomposition of polyurethanes’ soft segments [53]. These signals are noticeably separated for the reference foam. Similar to the hard segments, the presence of two peaks is attributed to the foams’ formulations. Nevertheless, the introduction of GTR particles, especially modified with oils, caused the shifts of these peaks and their overlapping. For GTR_20WO_ and GTR_40WO_, peak T_max3_ could not be distinguished. This effect was due to the overlapping of peaks characteristic for the decomposition of polyurethane soft segments and natural rubber present in the ground tire rubber particles [42]. The peak observed in the range of 448–457 °C (T_max5_) can be related to the thermolysis of organic residues from previous decomposition stages of polyurethane foam [90]. However, when the GTR was introduced, the magnitude of this peak was significantly enhanced. It was due to the degradation of styrene-butadiene rubber present in the structure of ground tire rubber.

Table 7 also presents the effect of GTR incorporation on the values of char residue. The experimental values (Exp.) were determined by thermogravimetric analysis, while theoretical ones (Theo.) were calculated according to the following Equation (8):

Theo. = (1 − 0.2) · Exp._PU_ + 0.2 · Exp._GTR_ (8)

where: Exp._PU_—experimental char residue of unfilled reference foam; Exp._GTR_—char residue for the particular type of GTR (Table 6). The 0.2 coefficient is associated with the content of GTR in composite foams (Table 3).

It can be seen that theoretical values of char residue for composite foams are higher than recorded experimental values. It can be attributed to the interfacial interactions, which decreased the stability of the polyurethane matrix. As suggested by the increasing values of sol fraction, the introduction of GTR particles, irrespectively of their type, resulted in the presence of unbound or loose macromolecules in the material, which may affect the stability of composites. Interestingly, the oil modification reduced the difference between theoretical and experimental values of char residue, which may suggest an improvement in interfacial interactions.

## 4. Conclusions

The presented paper aimed to analyze the influence of the thermo-mechanical treatment of ground tire rubber particles on the structure and performance of foamed PU/GTR composites based on a flexible polyurethane matrix. Applied GTR was modified in the reactive extrusion process. Moreover, except for the simple thermo-mechanical treatment addition of two types of rapeseed oil was investigated, fresh and waste—obtained as a by-product from the local restaurant. Introduction of oils into reactive extrusion of GTR caused the noticeable changes in filler properties, e.g., in the hydroxyl value. Independently of the treatment conditions, incorporation of modified GTR into polyurethane foams caused the reduction in average cell size, which could be attributed to the nucleating activity of filler and increased surface tension during foaming. The average cell diameter was reduced by 17–28%, which beneficially affected the performance of composites. Thermal insulation performance enhancement was noted, expressed by the 5% drop of thermal conductivity coefficient.

Changes in the cellular structure of foams also influenced the mechanical performance of analyzed materials. Generally, the presence of GTR particles caused deterioration of foam’s mechanical properties, despite the cell size reduction. Nevertheless, the incorporation of oils into the reactive extrusion of GTR was very beneficial. Compared to foam filled with thermo-mechanically treated GTR, samples containing GTR modified with waste oil showed compressive and tensile strength higher by 59–91% and 8–27%, respectively. The deterioration of the foams’ mechanical performance after the introduction of GTR was also affected by the decrease in glass transition temperature, which was relatively close to the ambient temperature during mechanical tests.

Moreover, the incorporation of ground tire rubber particles into flexible polyurethane foam was very beneficial in terms of thermal stability. The onset of thermal decomposition, determined as a temperature of 2 wt% mass loss, was shifted even by 13 °C towards higher temperatures for the composite containing thermo-mechanically treated GTR. Such an effect was attributed to the higher thermal stability of GTR compared to the polyurethane matrix.

In conclusion, the presented research work shows that the introduction of GTR may be considered a promising method for the improvement of the structure and performance of flexible polyurethane foams. Moreover, the use of the reactive extrusion process to thermo-mechanically modifies the GTR particles may significantly strengthen the interfacial interactions with the PU matrix, which results in beneficial changes in structure and performance of composites.

## Figures and Tables

**Table 1:** The list of materials used in the presented work.

Material	Producer	Properties/Additional Information
GTR modification		
Ground tire rubber	Recykl S.A. (Śrem, Poland)	Average particle size—0.6 mm
Fresh rapeseed oil	Zakłady Tłuszczowe Kruszwica S.A. (Kruszwica, Poland)	Fresh, unmodified oil
Waste rapeseed oil	Local restaurant (Gdańsk, Poland)	Post-consumer oil obtained after frying
Polyurethane foams preparation		
Rokopol®F3000	PCC Group (Brzeg Dolny, Poland)	Polyether polyol, propoxylated glycerol, hydroxyl value—53–59 mg KOH/g
Rokopol®V700	PCC Group (Brzeg Dolny, Poland)	Polyether polyol, propoxylated glycerol, hydroxyl value—225–250 mg KOH/g
Glycerol	Sigma Aldrich (Poznań, Poland)	Hydroxyl value—1800 mg KOH/g
SPECFLEX NF 434	M. B. Market Ltd. (Baniocha, Poland)	Polymeric methylenediphenyl-4,4′-diisocyanate, free isocyanate content—29.5%
PC CAT® TKA30	Performance Chemicals (Belvedere, UK)	Potassium acetate catalyst
Dabco33LV	Air Products (Allentown, USA)	Catalyst, 3 wt% solution of 1,4-diazabicyclo[2.2.2]octane in dipropylene glycol
Dibutyltin dilaurate	Sigma Aldrich (Poznań, Poland)	Organic tin catalyst
Distilled water	-	Chemical blowing agent
Determination of GTR hydroxyl value		
Acetone	Sigma Aldrich (Poznań, Poland)	Solvent
Dibutylamine	Sigma Aldrich (Poznań, Poland)	Analyte solution
Chlorobenzene	Sigma Aldrich (Poznań, Poland)	Solvent
Hydrochloric acid	Sigma Aldrich (Poznań, Poland)	Titrant
Toluene diisocyanate	Sigma Aldrich (Poznań, Poland)	Free isocyanate content—42%
3′,3″,5′,5″-Tetrabromophenol-sulfonphthalein	Sigma Aldrich (Poznań, Poland)	Indicator

**Table 2:** The properties of GTR samples applied in the presented work.

Sample	Oil Type and Content, phr	Screw Speed, rpm	Motor Load, %	SME, kWh/kg	TEC, kWh/kg	%_NCO_, %	Δ_NCO_, %	L_OH_ mg KOH/g
TM	-	50	33.0	0.053	0.160	36.3 ± 0.4	6.4 ± 0.4	41.1 ± 3.5
20FO	Fresh, 20	50	21.3	0.033	0.157	37.9 ± 0.5	4.8 ± 0.5	30.8 ± 3.2
40FO	Fresh, 40	150	4.6	0.008	0.165	36.7 ± 0.2	6.0 ± 0.2	38.3 ± 1.2
20WO	Waste, 20	50	16.4	0.026	0.157	32.4 ± 0.1	10.3 ± 0.1	66.6 ± 0.9
40WO	Waste, 40	150	4.7	0.008	0.168	29.9 ± 0.5	12.8 ± 0.5	82.3 ± 3.2

**Table 3:** Formulations applied during preparation of foams.

Component	Neat Foam	Composite Foams
	Content, wt%	
F3000	32.6	26.1
V700	32.6	26.1
Glycerol	0.8	0.6
DBTDL	0.6	0.5
33LV	0.4	0.3
TKA30	0.4	0.3
Water	0.3	0.3
pMDI	32.3	25.8
GTR/modified GTR	-	20.0
Isocyanate:hydroxyl ratio	1:1	

**Table 4:** The parameters of the cellular structure of prepared foams.

Parameter	Sample					
	PU	GTR_TM_	GTR_20FO_	GTR_40FO_	GTR_20WO_	GTR_40WO_
Average cell diameter, µm	308 ± 31	257 ± 32	241 ± 34	244 ± 36	238 ± 35	220 ± 33
Cell aspect ratio	1.46 ± 0.34	1.55 ± 0.42	1.50 ± 0.41	1.56 ± 0.42	1.49 ± 0.36	1.51 ± 0.40
Cell roundness	0.73 ± 0.15	0.70 ± 0.17	0.72 ± 0.17	0.69 ± 0.17	0.72 ± 0.17	0.71 ± 0.16
Open cell content, %	84.7 ± 0.2	84.3 ± 0.3	83.4 ± 0.6	83.1 ± 0.3	83.9 ± 0.4	83.4 ± 0.3
λ coefficient, mW/(m·K)	70.0 ± 0.1	66.0 ± 1.1	67.9 ± 1.0	69.0 ± 0.8	66.4 ± 1.5	66.8 ± 1.2

**Table 5:** The physico-mechanical properties of prepared foams.

Parameter	Sample					
	PU	GTR_TM_	GTR_20FO_	GTR_40FO_	GTR_20WO_	GTR_40WO_
Sol fraction content, %	2.3 ± 0.3	10.2 ± 0.3	16.0 ± 0.1	13.1 ± 0.2	9.4 ± 0.2	8.8 ± 0.3
Compressive strength at 50% deformation, kPa	45.1 ± 3.8	21.6 ± 1.3	17.7 ± 0.7	22.1 ± 2.0	34.4 ± 2.9	41.3 ± 1.8
Tensile strength, kPa	230 ± 6	171 ± 11	98 ± 5	126 ± 5	185 ± 8	217 ± 14
Elongation at break, %	195 ± 4	149 ± 11	121 ± 7	145 ± 14	162 ± 13	167 ± 2
Toughness, J/dm^3^	208 ± 18	117 + 23	60 ± 5	85 ± 10	145 ± 16	170 ± 15
Storage modulus at 22 °C, kPa	1358	226	195	427	534	1333
Tan δ at 22 °C	0.61	0.61	0.62	0.60	0.58	0.49
T_g_, °C	20.7	13.6	13.0	15.3	16.9	20.0

**Table 6:** The results of thermogravimetric analysis of GTR samples.

Sample	T_-2%_, °C	T_-5%_, °C	T_-10%_, °C	T_-50%_, °C	Char Residue, wt%	T_max1_, °C	T_max2_, °C
TM	260.0	308.5	347.4	443.9	37.48	377.9	436.6
20FO	269.3	315.7	346.2	427.0	32.24	382.1	441.6
40FO	280.2	323.3	349.8	417.5	27.72	389.1	441.6
20WO	263.0	305.6	339.8	426.6	32.53	381.5	440.6
40WO	265.5	307.5	339.6	415.6	27.49	386.1	440.6

**Table 7:** The results of thermogravimetric analysis of polyurethane/GTR composite foams.

Sample	T_-2%_, °C	T_-5%_, °C	T_-10%_, °C	T_-50%_, °C	T_max1_, °C	T_max2_, °C	T_max3_, °C	T_max4_, °C	T_max5_, °C	Char Residue, wt%		
										Exp.	Theo.	Difference
PU	215.0	253.5	308.2	372.5	187.7	227.6	346.7	393.4	456.8	13.2	-	-
GTR_TM_	228.0	283.4	316.7	390.9	193.3	232.8	347.8	399.1	449.8	16.3	18.1	−1.8
GTR_20FO_	228.4	283.9	318.7	391.3	190.4	232.1	348.7	398.3	449.4	16.0	17.0	−1.0
GTR_40FO_	226.6	278.8	318.1	389.0	189.7	230.3	351.6	393.3	448.2	15.0	16.1	−1.1
GTR_20WO_	220.0	277.6	314.8	390.0	183.4	221.4	-	390.7	449.4	15.7	17.1	−1.4
GTR_40WO_	214.7	272.2	316.3	390.3	182.4	218.3	-	392.5	449.1	14.6	16.1	−1.5

## Data Availability

Data is contained within the article. The data presented in this study are available in Ground Tire Rubber Filled Flexible Polyurethane Foam—Effect of Waste Rubber Treatment on Composite Performance.
